# Acute Aneurismal Bilateral Subdural Haematoma without Subarachnoid Haemorrhage: A Case Report and Review of the Literature

**DOI:** 10.1155/2014/260853

**Published:** 2014-06-18

**Authors:** Ossama Mansour, Tamer Hassen, Sameh Fathy

**Affiliations:** ^1^Neurology and Endovascular Neurology Department, Alexandria University Hospital, Champlion Street, Alazareeta, Medical Campus, Alexandria 23169, Egypt; ^2^Neurosurgery Department, Alexandria University Hospital, Champlion Street, Alazareeta, Medical Campus, Alexandria 23169, Egypt; ^3^Radiodiagnosis Department, Zagazig University Hospital, Zagazig 24533, Egypt

## Abstract

Spontaneous pure acute bilateral subdural haematoma (ASDH) without intraparenchymal or subarachnoid haemorrhage caused by a ruptured cerebral aneurysm is extremely rare. It can follow rupture of different aneurysms specially located in anterior incisural space; the most frequently encountered location is the PcoA aneurysms as demonstrated in the present case. We present a case report of a PcoA aneurysm presenting as pure bilateral ASDH. A high level of suspicion for bleeding of arterial origin should be maintained in all cases of acute subdural haematoma without history of trauma. The neurological status on admission dictates the appropriate timing and methodology of the neuroradiological investigations.

## 1. Introduction

In the majority of cases, acute subdural haematomas (ASDHs) are related to head trauma and are typically caused by disruption of superficial cerebral or cortical bridging veins. Spontaneous ASDHs are uncommonly encountered. Arteriovenous malformations [1], cocaine abuse [1], and many other causes have been proposed for this pathology [2]. Hemorrhage of aneurysms often presents as subarachnoid haemorrhage (SAH) and intracerebral haemorrhage (ICH); the ASDH is rare. ASDHs constitute neurosurgical emergencies and immediate treatment must be conducted before neurological deficits become irreversible.

We report a case of a spontaneous bilateral pure ASDH due to rupture of an aneurysm of the left posterior communicating aneurysm (PcoA) with reviewing of literature.

## 2. Case Report

**Figure 1:**
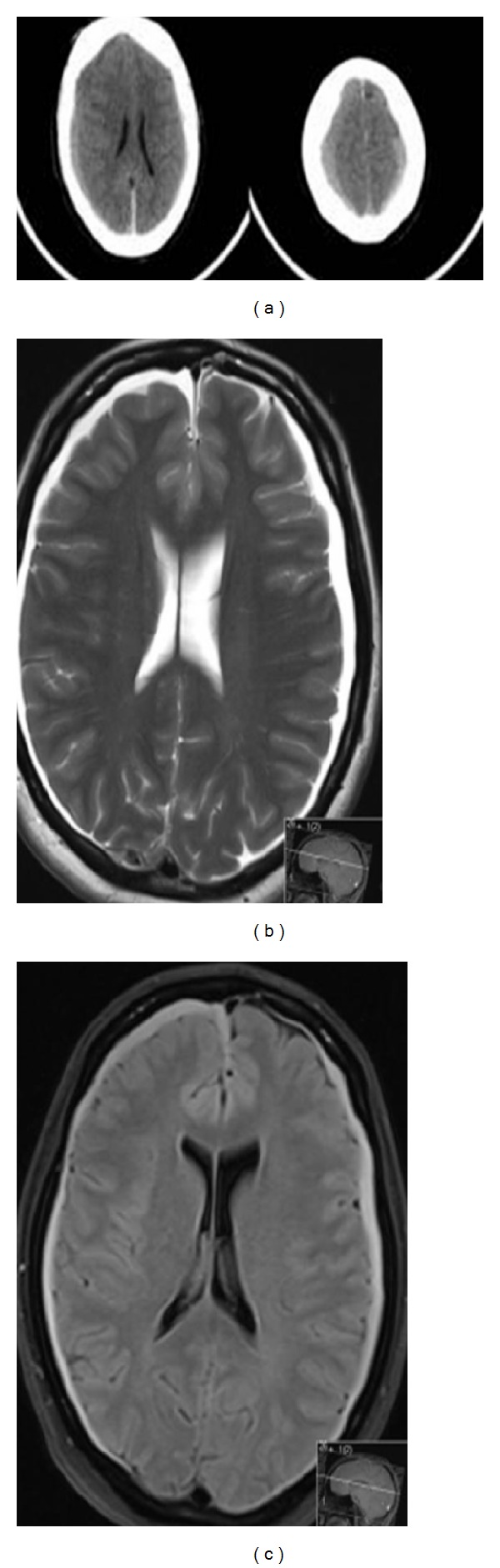
(a) Noncontrast head CT: bilateral hematomas are present. These are predominantly isodense to slightly hypodense compared to the adjacent gray matter (29–35 Hounsfield units), which could be explained by anemia (hemoglobin was 9 g/dL). (b) and (c) Bilateral subdural hematomas, confirmed as shown in both MRI T2 and FLAIR sequences.

A 51-year-old controlled diabetic and hypertensive man presented with a history of sudden onset of headache followed by reduction of the level of consciousness (GSC = 7) and right anisocoria, which could be old or just “false, localizing sign.” There was no history of trauma or previous neurological disease. CT scan revealed a bilateral high density subdural haematoma at the brain convexity without significant mass effect, SAH, or ICH (intracerebral hemorrhage); MRI confirmed the CT diagnosis (Figure 1). On admission, patient was somnolent and disoriented. There was no evidence of head injury and general physical examination was unremarkable. On admission, haemoglobin was 9 g/dL; other laboratory data including a coagulopathy screen and CSF analysis were normal.

**Figure 2:**
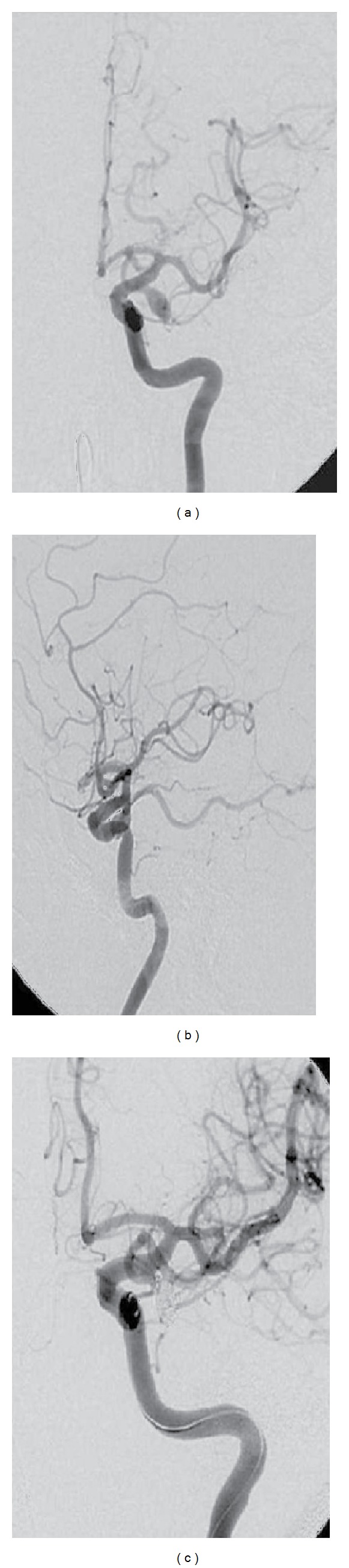
Left internal carotid DSA: AP (a) and lateral views, (b) demonstrating aneurysm of left PCoA, (c) AP view after embolisation (coils) showing complete obliteration of the aneurysm.

Owing to rapid clinical deterioration, he was intubated and taken to the operating room. Emergency bilateral frontotemporal craniotomy was performed and haematoma evacuated. There were neither signs of damaged underlying cortex nor typical signs of SAH on the surface. A bleeding cortical artery or other abnormalities could not be identified. On the first postoperative day, he recovered consciousness and was able to follow commands and to move all extremities. Because of the spontaneous course of the ASDH, he underwent cerebral angiography. This revealed a left saccular PCoA aneurysm (4.5 ∗ 7.5 mm) (Figures 2(a) and 2(b)). AP-view angiography showed an irregular shape of the aneurysm with additional small outpouching indicating possible point of rupture at the inflow zone (Figures 2(a) and 2(b)). The patient underwent successful coiling of the aneurysm (Figure 2(c)). His following hospital stay was uneventful, and he was discharged on the 11th postoperative day without neurological deficits, being able to return to his normal life.

## 3. Discussion

ASDHs develop spontaneously in patients without history of trauma or coagulopathy and an aneurysm rupture is responsible for the majority of such cases with incidence of about 0.5% to 7.9% [3–6].

**Figure 3:**
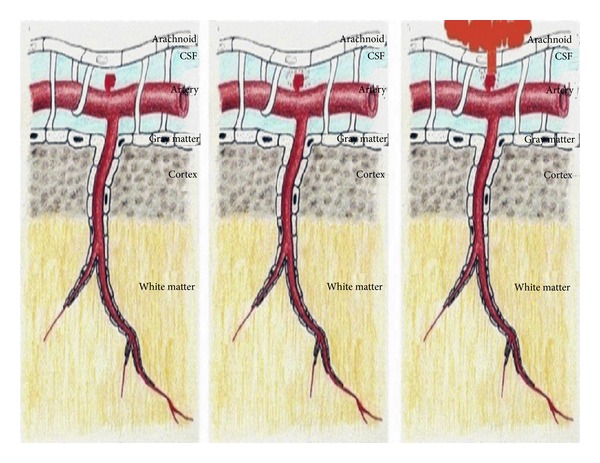
Diagram illustrating the different stages that possibly make an aneurysm bleed in subdural space.

Several mechanisms have been proposed to explain the occurrence of ASDH after aneurysm rupture. Firstly, successive minor sentinel haemorrhages may fix an aneurysm to local arachnoid adhesions (Figure 3) resulting in bleeding directly into the subdural space when an arachnoid tear occurs after aneurysmal rupture or simply through a weak point at the arachnoid membrane without previous sentinel bleeding [6]. A second mechanism may be due to a haemorrhage under high pressure, leading to pia-arachnoid rupture and extravasation of blood into the subdural space, where in this scenario the subdural hematoma may develop secondary to the compensatory decompression of an intracerebral hematoma into the subdural space following disruption of the arachnoid covering the cerebral cortex [6].

Biesbroek et al. reported retrospectively on 1757 ruptured aneurysms where 63 cases had an ASDH (as a presenting manifestation). Increasing age, sentinel headache, ICH, and aneurysms at the PCoA were independent risk factors for ASDH. Patients with a basilar or vertebral aneurysm have a low risk for ASDH [4]. The incidence of pure ASDH, without associated ICH or SAH, due to a ruptured aneurysm is extremely rare. The reported cases in literature are summarized in Table 1. The most frequent site of aneurysm causing pure ASDH was at the origin of the PCoA from the internal carotid artery (IC-PC) (60% of the cases), followed by the distal anterior cerebral artery (ACA) (16%) and middle cerebral artery (12%).

In the present case, the bilateral ASDH due to a PCoA aneurysm presented as pure bilateral ASDH. Anatomically, the anterior incisural space, which is located anterior to brainstem, contains the posterior communicating artery (PCoA), anterior choroidal artery, and basilar bifurcation; additionally, it contains the supraclinoidal portion of the internal carotid artery [4]. This space opens laterally into the part of the Sylvian fissure situated below the anterior perforated substance [4]. This explains the occurrence of a subdural haematoma following aneurysm rupture arising from arteries located in this space like PCoA aneurysm as in the present case, where blood finds its way through the abovementioned pathway to the subdural spaces.

Pure ASDH following rupture of intracranial aneurysm carried a poor prognosis in 34.3% (12 of 357; 14.3% disabled and 22.8% died due to bleeding) of the reported cases in literature. The 22.8% mortality rate in this group of patients is slightly higher than the mortality rate of simple traumatic subdural haematomas (reported to be 20%) [4]. The simple traumatic ASDHs are distinguished from the complicated traumatic subdural haematomas by the absence of parenchymal damage [4]. This assumed that the difference in mortality rate may be due to the initial elevated intracranial pressure caused by the subdural haematoma or by rebleeding of the aneurysm before its occlusion. Therefore, adequate diagnostic investigations and respective prompt treatment are essential for a better outcome. If the patient presents with a stable neurological condition, angiography should be performed prior to surgery to dictate the best strategy. In the presence of a definite bleeding source, emergency surgery should be adopted to evacuate the haematoma and operate on the bleeding source.

If the angiography does not demonstrate the source of bleeding, the patient can be managed conservatively or surgically according to the subsequent evolution of the neurological status. In cases of patients presenting with rapid neurological deterioration, immediate decompression surgery should be performed before performing angiography. In the absence of intraoperative identification of a cortical arterial rupture or other source of bleeding, complementary postoperative arteriography is required, to rule out sources which could not be detected during surgical evacuation.

In summary, a high level of suspicion for bleeding of arterial origin should be maintained in all cases of ASDH without history of trauma which may mandate vascular assessment as routine.

## Figures and Tables

**Table 1:** Cases of pure subdural haematoma (without subarachnoid haemorrhage and without intraparenchymal haematoma) caused by rupture of intracranial aneurysm [5, 6].

Case	Author	Age (years)	Sex	Symptoms/signs	Location of aneurysm	Location of subdural haematoma	Treatment	Outcome
1	Rengachary et al. (1981) [7]	49	M	Confusion and dysphasia	Sylvian branch of MCA	Convexity	Haematoma evacuation and clipping	Good
2	Eggers et al. (1982) [8]	34	F	Headache	IC- PC	Convexity	Haematoma evacuation	Good
3	Williams et al. (1983) [9]	18	F	Coma	IC-PC	Convexity	Haematoma evacuation and clipping	Disabled
4	Friedman et al. (1983) [10]	55	F	Headache	IC-PC	Tentorium and interhemispheric	Clipping	Good
5	O'Leary et al. (1986) [11]	28	F	Coma	MCA	Convexity	None	Dead
6	Kondziolka et al. (1988) [12]	43	M	Coma	IC-PC	Tentorium and convexity	Haematoma evacuation and clipping	Good
7	Kondziolka et al. (1988) [12]	38	F	Coma	IC-PC	Tentorium and convexity	Haematoma evacuation and clipping	Disabled
8	Shinmura et al. (1989) [13]	44	F	Coma	MCA	Convexity	Haematoma evacuation and clipping	Disabled
9	Onda et al. (1989) [14]	51	F	Semicoma	IC-PC	Convexity	Haematoma evacuation and clipping	Disabled
10	Watanabe et al. (1991) [15]	27	M	Semicoma	Distal ACA	Interhemispheric and convexity	Haematoma evacuation and clipping	Dead
11	Ragland et al. (1993) [16]	55	M	Coma	AcomA	Convexity	Haematoma evacuation	Dead
12	Hatayama et al. (1994) [17]	55	M	Semicoma	Distal ACA	Interhemispheric and convexity	Haematoma evacuation and clipping	Good
13	Hatayama et al. (1994) [17]	66	F	Semicoma	Distal ACA	Interhemispheric, convexity, and tentorium	Haematoma evacuation and clipping	Disabled
14	Ishibashi et al. (1997) [18]	54	F	Headache	IC	Tentorium and convexity	Haematoma evacuation and clipping	Good
15	Satoh et al. (1999) [19]	58	F	Semicoma	IC	Convexity	Haematoma evacuation and clipping	Good
16	Satoh et al. (1999) [19]	25	F	Headache	IC	Convexity	Haematoma evacuation and clipping	Good
17	Satoh et al. (1999) [19]	22	F	Coma	IC	Convexity	Haematoma evacuation and clipping	Good
18	Nonaka et al. (2000) [20]	52	F	Coma	IC	Tentorium and convexity	Haematoma evacuation and clipping	Good
19	Ishikawa et al. (2000) [21]	62	M	Headache and ptosis	IC	Tentorium and interhemispheric	Clipping	Good
20	Inamasu et al. (2002) [22]	28	F	Coma	IC	Convexity	Haematoma evacuation	Dead
21	Araki et al. (2002) [23]	55	F	Headache, ptosis, and semicoma	IC	Convexity	Haematoma evacuation and clipping	Good
22	Blake et al. (2003) [24]	35	F	Coma	IC	Convexity	Non	Dead
23	Katsuno et al. (2003) [25]	62	F	Headache, nausea, and dizziness	Distal ACA	Interhemispheric and convexity	Haematoma evacuation and clipping	Good
24	Shenoy et al. (2003) [26]	45	F	Headache and blurring of vision	MCA	Convexity	Haematoma evacuation and clipping	Good
25	Shenoy et al. (2003) [26]		F	Semicoma and hemiparesis	IC-PC	Convexity	Haematoma evacuation and clipping	Good
26	Koerbe et al. (2005) [27]	63	F	Headache and semicoma	Bifurcation of ICA	Convexity	Hematoma evacuation and coiling	Good
27	Boujemâa et al. (2006) [28]	44	F	Coma	IC-PC	Bilateral convexity and a hyperdensity on the tentorium cerebelli	Hematoma evacuation and coiling	Dead
28	Gilad et al. (2007) [29]	47	M	Nausea and vomiting	AcomA	Sella, migrating to spinal canal	Coiling	Good
29	Kocak et al. (2009) [30]	47	F	Not described	AcomA	Not described	Clipping	Good
30	Weil et al. (2010) [31]	51	F	Coma	MCA	Convexity	Haematoma evacuation and coiling	Dead
31	De Blasi et al. (2010) [32]	47	F	Headache and stupor	ICA-PcomA	Convexity	Coiling	Good
32	De Blasi et al. (2010) [32]	60	F	Headache and abducens palsy	MCA	Convexity	Clipping	Good
33	Takada (2012) [33]	54	M	Headache	AcomA	Tentorium and convexity	Clipping	Good
34	Mrfka (2012) [6]	40	F	Headache, nausea, and vomiting	PcomA	Convexity	Haematoma evacuation and coiling	Good
35	Jie Gong (2014) [5]	43	M	Headache	MCA	Convexity	Haematoma evacuation and resection	Good
